# Pulsatile-flow culture: a novel system for assessing vascular-cell dynamics†

**DOI:** 10.1039/d4lc00949e

**Published:** 2025-02-24

**Authors:** Neda Salimi-Afjani, Robert Rieben, Dominik Obrist

**Affiliations:** a Department for BioMedical Research, University of Bern Murtenstrasse 28 3008 Bern Switzerland neda.salimiafjani@unibe.ch; b Graduate School for Cellular and Biomedical Sciences Mittelstrasse 43 3012 Bern Switzerland; c ARTORG Center for Biomedical Engineering Research, University of Bern Freiburgstrasse 3 3010 Bern Switzerland

## Abstract

We describe a model system for vascular-cell culture where recirculating fluid flow in standard culture plates is generated by gravity using a combination of platform tilt and rotation (nutation). Placed inside a cell-culture incubator, variable nutation speeds provide pulsatile shear stresses to vascular cells within the physiological range. The effect of these stresses on cells is demonstrated here using standard laboratory techniques such as immunofluorescent staining, immunoblot, and supernatant analyses. This gravity-driven model framework is well-suited for assessing dynamic conditions for mono- and co-cultures. In addition, the modular design and the use of off-the-shelf components make the system economical and scalable.

## Abstract 2

A simple, scalable, economical and well-characterized system for investigating vascular mural cells under physiological conditions with pulsatile shear stress, advancing vascular research and translational studies.

## Graphical abstract

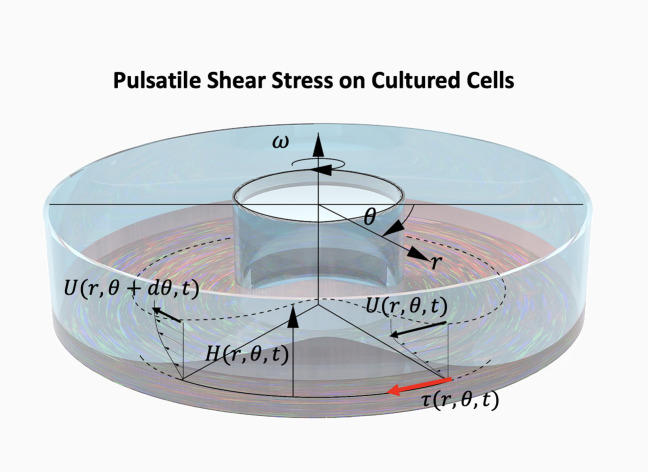

## Introduction

Endothelial cells line the luminal surface of the vascular tree and are constantly subjected to shear stress exerted by pulsatile blood flow and the influence of underlying smooth muscle cells.^1^ The endothelial cells respond to flow-induced shear stress through surface molecules which transfer shear stress to the cytoskeleton, and the result is stress-filament contraction and cellular alignment in the direction of flow.^2^ In addition, the interaction between blood flow and endothelial cells is responsible for regulating vascular tone through the production of the vasoactive molecule nitric oxide.^3^ For model investigations of endothelial cells in health and disease, it is important that the experimental conditions be as close to natural as possible so that shear stresses resulting from fluid flow play a decisive role.

Microfluidic devices with confined channels are often used to model blood vessel features.^4^ For cell-culture systems using such microchannels, the liquid culture medium is typically driven by an external pump. This medium is responsible for providing oxygenation and physiological shear stress stimuli to the cells lining the walls of these closed systems. However, flow pulsatility (*e.g.*, induced by a peristaltic pump) is often diminished by the compliance of the tubing that connects the pump to the microfluidic device and by the presence of bubble traps, such that cells may not experience physiological mechanical stimuli.^5^ Moreover, cells can also experience impaired flow environments due to channel geometry and proximity to the entry/exit ports. The design of microfluidic devices is also constrained by limitations of micro-fabrication processes such as photolithography, micromolding, polymerization of cell- and microscope-friendly silicone-based liquids for microchannel fabrication, and the position of ports for fluid entry/exit.^6^ In addition, each microchannel can only accommodate a small fraction of the cells normally found in a standard cell-culture dish. This small number of cells is often inadequate for downstream functional assessments such as flow cytometry and immunoblotting, even with experiment-pooling strategies. Finally, the small size and confined nature of the microchannels limit manual and optical access to this type of cell culture.^7^ Recently, attempts have been made to replace external flow pumps for micro-channels with orbital oscillatory and nutating mixers.^8,9^ Despite detailed analysis of the underlying mechanics of the sloshing fluid flow,^10,11^ in-depth functional assessments of such cell-culture systems are still lacking.

To address the limitations of microfluidic devices outlined above, a pumpless and tubeless recirculating flow-culture system was developed using readily available tissue-culture components. The system's functionality was assessed using cultured mammalian cells isolated from blood vessels. This compact open-channel system can be operated continuously inside a standard cell-culture incubator with gravity providing the driving force for recirculating pulsatile flow using a fixed-angle nutating mixer. The physiologically relevant flow-induced shear stress on the cell bed (bed shear stress) can be regulated by changing the rotation speed of the platform. The circular flow in this system also avoids the problem of distinct environments for cells located at different positions along channels with finite length, yielding more homogeneous results.

## Results

### A. Flow characterization of the model

#### a. Traveling waves and velocities

**Fig. 1:**
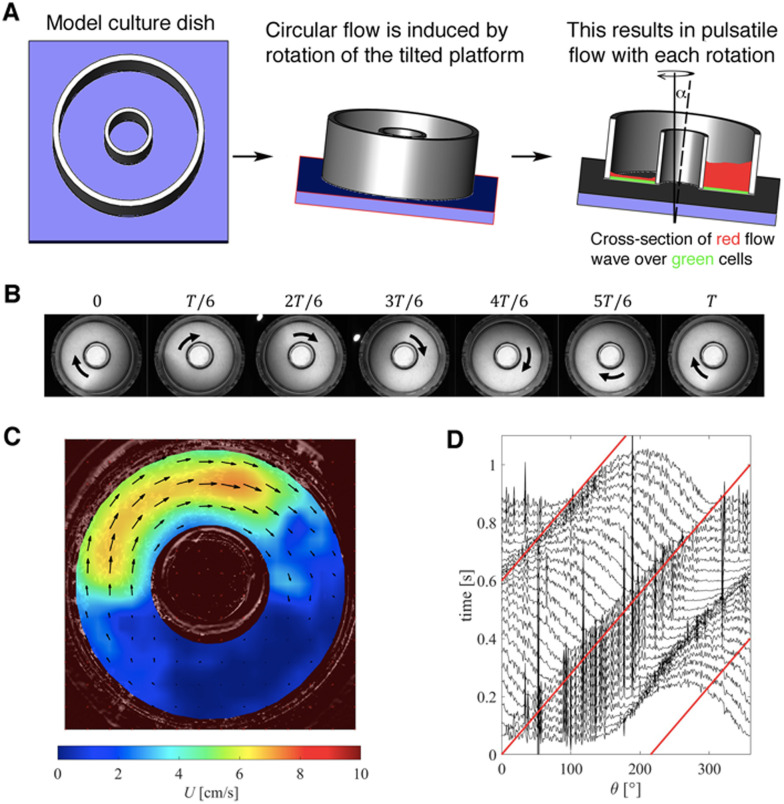
(A) Explanatory images of pulsatile-flow culture showing the influence of tilt + gravity + rotation on fluid flow over cultured cells in a well. (B) Video frames of a well containing dye solution that shows a travelling wave over a complete period T (the white spots in the frames for 2T/6 and 3T/6 are laser indicators used for video synchronization with tilt position). (C) PIV analysis of the same configuration indicating circular streamlines with peak velocities of approximately 7 cm s^−1^. (D) Representations of fluid layer thickness for consecutive video frames plotted against the azimuthal angle *θ*. The red lines correspond to an angular wave speed of 2π [rad s^−1^]. All results were obtained using 60 rpm, *μ* = 1 mPa s^−1^ and *V*_0_ = 1.5 ml.

The rotatory tilt motion (Fig. 1A) of the nutating mixer induced swirling flow in the well moving in the direction of the tilt motion of the mixer (Fig. 1B and Movie S1†). This flow had the nature of a traveling-wave, forming a wave crest (thicker fluid layer) that moved with constant angular wave speed in a clockwise direction. Particle image velocimetry (PIV) measurements on the fluid surface showed velocity vectors pointing predominantly in the direction of wave propagation and forming nearly circular streamlines (Fig. 1C). Wave crests showed higher velocities, while the fluid was nearly at rest in the wave troughs. Precise velocity measurements were difficult in these trough areas because the surface particles used for PIV tended to accumulate at the wave crest and were, therefore, very sparse in these areas.

**Fig. 2:**
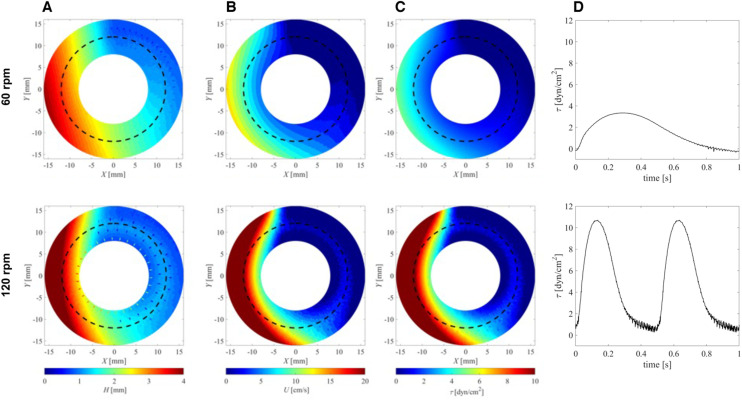
Examples of flow-culture dynamics at two different mixer speeds for the high viscosity medium (*μ* = 2 mPa s). (A) Heat maps of fluid layer thickness *H*. (B) Heat maps of surface velocity *U*. (C) Heat maps of bed shear stress fields *τ*. (D) Wave form of bed shear stress at a radius of 12 mm (corresponding to dashed circular line in A–C).

PIV velocity field measurements (Fig. 1C) agreed well with the velocity fields derived from fluid layer thickness measurements (Fig. 2B). The differences observed close to the inner/outer well walls were probably associated with measurement uncertainties of the PIV method in those regions. In addition, secondary flow phenomena in the wall regions led to radial flow components that were neglected in the calculations of flow velocities from fluid layer thickness.

In the middle region of the open channel (*r* = 12 mm), peak fluid velocities ranged from 9.7 cm s^−1^ (60 rpm) to 20.4 cm s^−1^ (120 rpm). Experiments with reduced viscosity of the culture medium (*μ* = 0.001 Pa s) led to minor increases in peak velocities (10.0 cm s^−1^ for 60 rpm, 20.5 cm s^−1^ for 120 rpm), suggesting only a minor influence of viscosity on flow velocities.

Traveling-wave speeds were measured by analyzing fluid layer thicknesses along a concentric circular line (*r* = 12 mm) in consecutive video frames (Fig. 1D). The red line in Fig. 1D illustrates an angular wave speed of *ω*_p_ = 2π [rad s^−1^], which agrees well with the change of the layer thickness over time and matches the nutating mixer frequency of 60 rpm. Wave speeds were also evaluated for other radii and at different mixer frequencies. We found that angular wave speeds matched mixer frequencies for all configurations.

The traveling wave yields a pulsatile flow in the annular channel of the well with pulsation frequencies of *ω* = 2π⋯4π [rad s^−1^]. Such pulsatile flows are associated with Stokes boundary layers at the bed of the well. According to equation
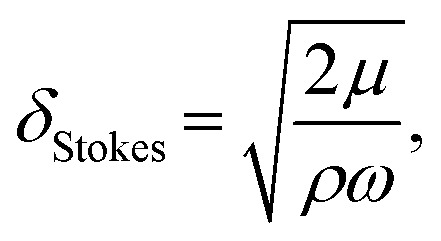
the given pulsation frequencies yield Stokes boundary layer thicknesses *δ*_Stokes_ of 0.40 to 0.56 mm. This was generally smaller than the observed fluid layer thickness at the wave crest, where the maximum bed shear stresses were expected. Therefore, we assumed in the following analysis that the bed shear stress was governed by equation
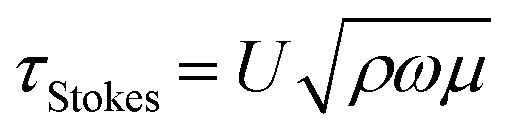
The formula for the bed shear stress due to a parabolic flow profile
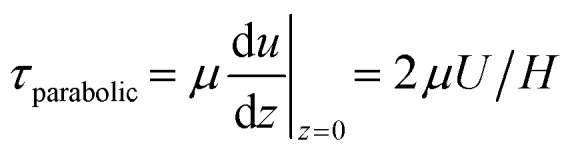
would only apply if the fluid layer was thinner than the theoretical Stokes boundary layer thickness which was not the case in our experiments.

#### b. Fluid layer thickness

Fig. 2A shows fluid-layer thicknesses (*Ĥ*) for 60 and 120 rpm mixer speeds. The measured mean fluid layer thickness was 1.8 to 2.1 mm, which agrees well with the filling height for 1.5 ml of culture medium. Azimuthal variations in fluid layer thickness were directly related to fluid velocities (see Methods), such that wave-crest height increased with increasing fluid velocities (Table 1). At no well location was fluid-layer thickness less than 0.8 mm, so cultured cells were always covered with fluid.

**Table 1:** Maximum bed shear stresses experienced by cells located at *r* = 12 mm

Viscosity *μ* [Pa s]	rpm	*H*_max_ [mm]	*U*_max_ [cm s^−1^]	*τ*_max_ [dyn cm^−2^]
0.001 (low)	60	3.7	10.0	2.5
	120	3.8	20.5	7.3
0.0021 (high)	60	3.6	9.7	3.5
	120	3.9	20.4	10.5

#### c. Bed shear stress

Fig. 2C shows instantaneous bed shear stresses (BSS) *τ* for different configurations (calculated from 
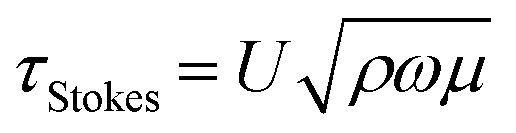
) computed from the fluid surface velocity fields *U* shown in Fig. 2C. Based on the observation that the surface velocities were predominantly directed azimuthally, we assumed the same for the bed shear stresses. These BSS fields rotated with angular wave speeds *ω*_p_ such that cells cultured on the bottom experienced pulsating stresses.

The waveforms of these stresses are shown in Fig. 2D for cells located in the center of the fluid channel (*r* = 12 mm). They were extracted from the stress fields in Fig. 2D by extracting values along a concentric circle in counter-clockwise direction (because the wave travels in a clockwise direction).

These waveforms were reminiscent of pulsatile profiles of arterial blood flow with peak shear stresses ranging from 2.5 dyn cm^−2^ to 10.5 dyn cm^−2^, with higher values for higher mixer speeds and for higher fluid viscosities. These results are summarized in Table 1.

### B. Biological validation of the model

#### a. Effect of pulsatile flow on cell alignment

**Fig. 3:**
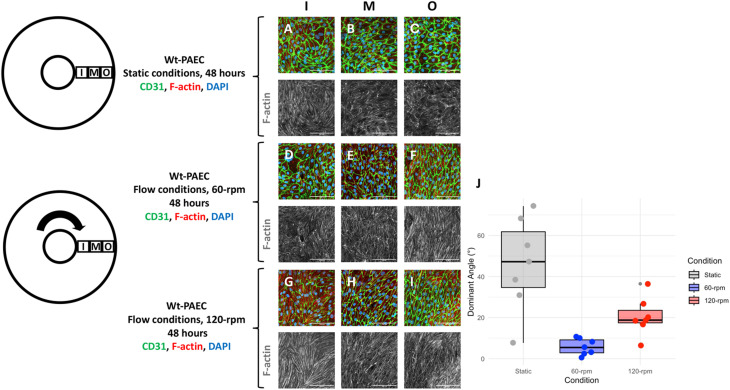
Expression of CD31 and F-actin in wild-type porcine aortic endothelial cells under different flow conditions. (A–C) No flow, static conditions; (D–F) 60 rpm flow for 48 hours; (G–I) 120 rpm flow for 48 hours. Composite images with immunofluorescence staining for CD31 (green) and F-actin filaments (red), as well as cell nuclei stained with DAPI (blue) are shown in the top rows for each condition. To visualize cell alignment more clearly, F-actin staining is also shown as original grayscale images just under the corresponding composite images. Each set of images shows cells from different flow-channel regions (I = inner, M = middle, O = outer) as identified on the drawings of the flow channels to the left. Scale bars = 100 μm. (J) Dominant F-actin orientation *versus* flow direction for all three flow conditions. F-actin showed no specific alignment under static conditions, whereas both 60 and 120 rpm conditions aligned almost parallel with flow.

Endothelial cells are often recognized for their ability to align in the direction of fluid flow, a morphological adaptation that serves as an indicator of their healthy response to flow and shear stress.^12^ Cell alignment (under both static and flow conditions) was assessed using 0, 60, and 120 rpm conditions for 48 hours. Cultured wild-type porcine aortic endothelial cells (wt-PAEC) were assessed for the expression of the adhesion–junction molecule CD31 and for F-actin, a cytoskeletal filament marker. Under all conditions, cells were positive for CD31 and there was homogeneous coverage of the channel by a monolayer of endothelial cells (Fig. 3). Under static conditions, cells had cobblestone-like appearance and cytosolic F-actin filaments were located peripherally around cell nuclei (Fig. 3A–C). In contrast, endothelial cells were spindle-shaped under flow conditions and F-actin filaments were aligned towards the direction of flow (Fig. 3D–I). Under both flow conditions, the F-actin filaments predominantly aligned nearly parallel to flow direction, with angles ranging between 0° and 30° (relative to the streamlines). At 120 rpm, the alignment of F-actin filaments at each observed location (*n* = 7) within the channel cross-section exhibited slight variations (Fig. 3J), while still remaining within this angular range. These differences in alignment may be attributed to greater variation in radial bed-shear stresses (Fig. 2 and S2†). Notably, the number of filaments aligned within this angular range at each radial position showed lower dispersion, suggesting a higher degree of alignment in that direction (Fig. S3†). In contrast, at 60 rpm, where the radial bed-shear stress was more homogeneous (Fig. 2 and S2†), the dominant alignment angle was almost parallel to the flow direction across the channel cross-section (Fig. 3 and S3†). However, these filaments exhibited greater dispersion, highlighting the influence of lower shear stress on cytoskeletal alignment.

#### b. Effect of pulsatile flow on eNOS production

**Fig. 4:**
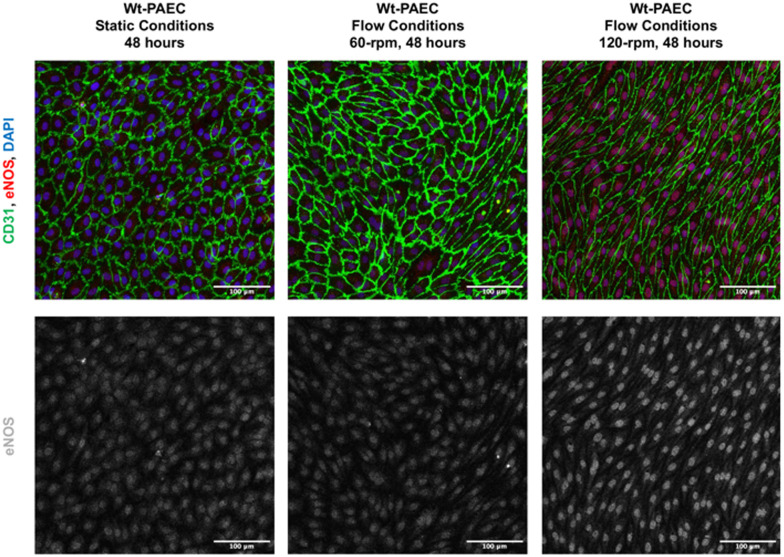
eNOS immunofluorescent staining in porcine aortic endothelial cells under static and flow conditions. DAPI = blue, CD31 = green, and eNOS = red. For clarity, the original greyscale representation of eNOS expression in the lower panels was observed in almost all endothelial cells, but labelling intensity was higher under flow conditions. Scale bars = 100 μm. Contrast and brightness of immunofluorescence images was uniformly adapted to increase visibility by eye.

**Fig. 5:**
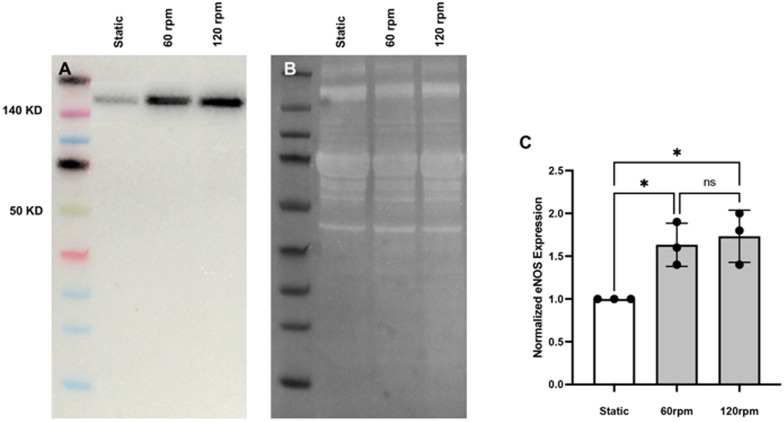
Western-blot analysis of eNOS protein levels in porcine aortic endothelial cells under static and flow conditions. (A) Composite representative image showing molecular weight markers and eNOS protein expression using 5 μg aliquots of lysates from endothelial cells incubated under either static or flow (60 and 120 rpm, 48 hours) conditions. Representative total protein analysis (B) of the same lanes was then used to normalize the eNOS band intensities. (C) eNOS protein expression in endothelial cells cultured under flow conditions compared to static. Data are mean ± SD of normalized quotients, *N* = 3 static, *N* = 3 flow, one-way ANOVA. * = *p* < 0.05.

Endothelial nitric oxide synthase (eNOS) is an enzyme responsible for nitric oxide (NO) production. NO is known to play a fundamental role in regulating vascular tone in mammals.^13^ It acts as a signaling molecule that communicates with underlying smooth muscle cells to adjust the diameters of blood vessels and, by this, helps to regulate both systemic and regional blood flow and pressure.^14^ Measuring the expression level of eNOS is an indirect way of assessing possible NO production, so we assessed eNOS expression levels in wt-PAEC under different flow conditions. eNOS immunofluorescent staining increased under flow conditions compared to static conditions (Fig. 4), so further immunoblotting analyses were performed to compare endothelial eNOS protein expression levels under both conditions. As shown in Fig. 5, eNOS protein expression was significantly higher under both flow conditions compared to static conditions, mirroring the immunofluorescence results.

#### c. Expression of endothelial glycocalyx in pulsatile-flow culture

**Fig. 6:**
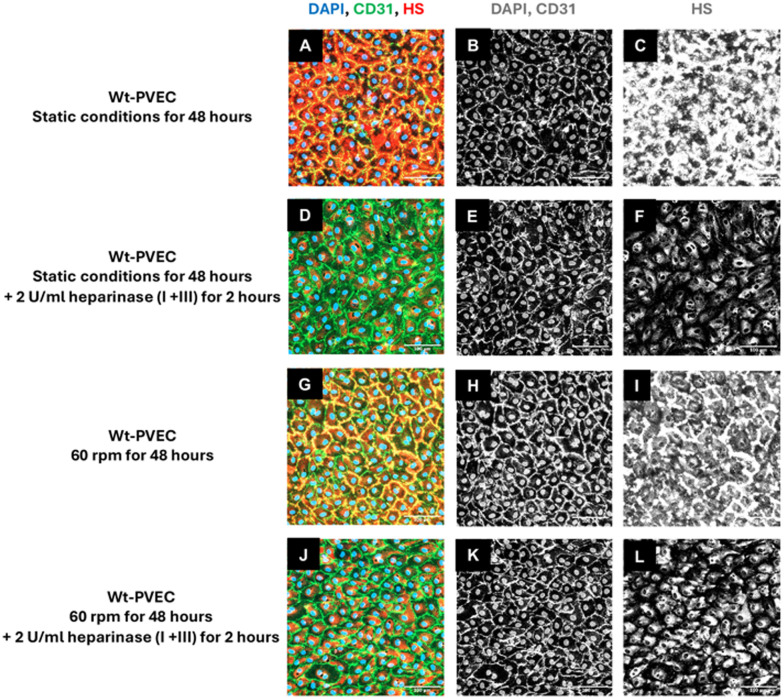
Expression of heparan sulfate (HS) in porcine venous endothelial cells under static and flow conditions, both after 48 hours. (A–C) No flow. (D–F) Heparinase I + III + no flow conditions. (G–I) Flow conditions. (J–L) Heparinase I + III + flow conditions. Composite images with immunofluorescence staining for CD31 (green) and heparan sulfate (red), as well as cell nuclei stained with DAPI (blue), are shown in the left column for each condition (A, D, G, J). CD31 (B, E, H, K) and heparan sulfate (C, F, I, L) staining of each condition are also shown in gray scale for clarity in the second and third columns of the respective conditions. Scale bars = 100 μm.

The glycocalyx is a protective outer layer for endothelial cells. It is a coating matrix that features proteoglycans and glycosaminoglycans covering the surfaces of endothelial cells. In blood vessels, the luminal glycocalyx plays a pivotal role in the endothelial cell responses to environmental factors,^15^ so its dynamic role in vascular studies is highly relevant. We assessed the formation of heparan sulfate (HS), one of the most abundant glycosaminoglycans in the glycocalyx, under both static and flow conditions using immunofluorescent staining. Wild-type porcine venous endothelial cells (wt-PVEC) were subjected to either static or their *in vivo*-like low flow conditions (60 rpm) for 48 hours. In addition, apical HS was specifically cleaved from the protein backbone of endothelial cells using 2 U ml^−1^ of heparinase III and I as a control. As shown in Fig. 6, HS was expressed intracellularly and apically on these endothelial cells under static and flow conditions (Fig. 6A and G). The co-localization of apical HS (red) with CD31 (green) was registered as yellow. The expression of apical HS but not the intracellular HS was diminished under both conditions by treatment with heparinase I and III (Fig. 6D and J).

### C. Under flow, both co-culture and supernatant assessments are possible

#### a. Co-culture of endothelial and smooth muscle cells on a membrane

**Fig. 7:**
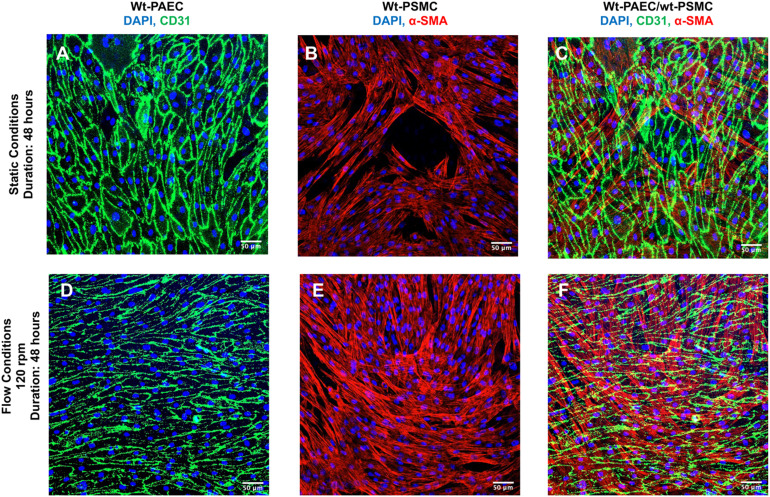
Two-dimensional projections of *z*-stack fluorescence images of co-cultured autogenic wild-type porcine aortic endothelial cells and porcine smooth muscle cells separated by a thin, porous, transparent polyester membrane at different flow conditions. Porcine aortic endothelial cells positively stained for marker molecule CD31 (A and D) and smooth muscle cells, positively stained for marker molecule alpha-smooth muscle actin (α-SMA) (B and E) their composite images (C and F) under static conditions (upper raw: A–C) and flow conditions (lower raw: D–F). Scale bars = 50 μm.

Endothelial cells line the inner surface of blood vessels, and they orient themselves along the direction of blood flow. Smooth muscle cells are organized in layers around the vessel, perpendicular to the direction of blood flow. This orientation allows cells to contract or relax efficiently, controlling vessel diameter and blood pressure. This alignment also minimizes the shear stress on individual cells.^16,17^ Porcine aortic endothelial cells and smooth muscle cells from the same animal were seeded over 24 hours on opposite sides of a precoated porous membrane. Each membrane was stored under static conditions overnight to allow both cell types to adhere. The membranes were then exposed to either static or flow (120 rpm) conditions for an additional 48 hours. After immunofluorescent staining, the positioning (relative to each other) and morphologies of the two cell types were assessed using confocal microscopy *z*-dimension stacks. Sample 2D projections of the 3D *z*-stacks were then made for each membrane (Fig. 7). Under static conditions, the endothelial cells had a cobblestone shape and the smooth muscle cells appeared almost parallel to the endothelial cells (Fig. 7B). However, under flow conditions, the endothelial cells aligned toward the flow direction but smooth muscle cells were almost perpendicular to the flow direction and to endothelial cell orientation (Fig. 7E).

**Fig. 8:**
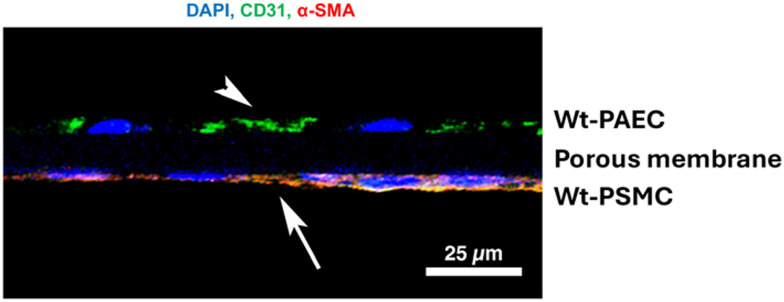
Cross-section of wild type porcine endothelial cells and wild type smooth muscle cells co-cultured on a porous membrane after 48 hours of flow at 120 rpm. Endothelial cells were stained for the expression of their marker molecule CD31 (arrowhead, green) and smooth muscle cells for their marker molecule, alpha-smooth muscle actin (arrow, red), along with cell nuclei (blue). CD31-positive endothelial cells are above the transparent membrane in the image with smaller cell nuclei and smooth muscle cells with elongated cell nuclei are positive for cytoskeletal actin. Scale bar = 25 μm.

In separate experiments, membranes with co-cultured cells were fixed, snap-frozen, and cut in cross-section using a cryostat (12 μm, CM 3050, Leica Biosystems, Deerpark, USA) before staining for CD31 and α-SMA. Fig. 8 shows a cross-section of a membrane after staining both cell types on opposite sides of it for their marker molecules.

#### b. Molecular quantifications of the supernatant

In pump-driven systems, relatively large media volumes are often required. In this flow system, the 1.5 ml of medium recirculates within each channel. This difference in analyte dilution makes supernatant analyses possible in pulsatile-flow culture not only due to the increased number of cells, but also to a higher analyte signal-to-noise ratio.

**Fig. 9:**
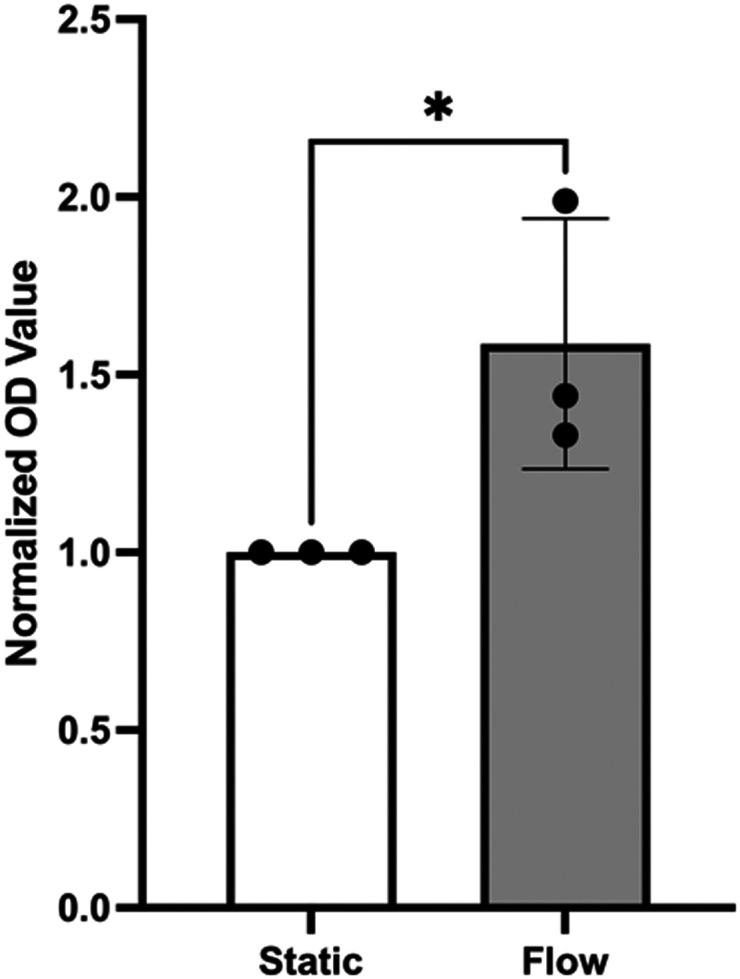
Supernatant analysis for porcine aortic endothelial cell viability under static and flow conditions. Flow led to almost twice the formazan formation compared to static conditions. The formazan concentration in each condition was measured at 450 nm and the data normalized as an index to the static baseline. Data are mean ± SD of normalized quotients, *N* = 3 static, *N* = 3 flow, unpaired *t*-test. * = *p* < 0.05.

Assays that use cell-culture supernatants to quantify mitochondrial dehydrogenase activity as an indicator for cell viability are often used to assess overall cell health. This is because mitochondrial oxidative phosphorylation can reduce a colorless tetrazolium salt introduced into the supernatant to red formazan that can be measured by absorbance.^18^ Using this assay, the flow medium was used to examine the influence of flow on the viability of wt-PAEC in pulsatile-flow culture using absorption quantification of red formazan formation in the supernatant. As shown in Fig. 9, pulsatile flow led to a significantly higher formation of formazan than no flow condition.

## Discussion

Numerous *in vitro* systems have been developed to study endothelial cells under flow conditions, yet they often face limitations, particularly regarding the type of flow they can simulate. Most models are constrained to steady or fixed-pulse frequency flow, whereas endothelial cells *in vivo* are exposed to varying blood flow and shear stresses.^19^ These variations in shear stress have a direct impact on endothelial cells. For example, variable pulsatile flow, as opposed to steady flow, enhances the production of eNOS for regulation of vessel tone in response to blood flow changes.^20^ The present pulsatile-flow culture inherently features variable pulsatile flow (Fig. 2). And, as demonstrated in Fig. 5, eNOS expression significantly increased in endothelial cells under flow conditions compared to static conditions.

Variable pulsatile flow can also be achieved in an open channel using an orbital oscillatory shaker which drives the flow by centripetal acceleration, calculated as *R*_O_*ω*^2^ where *R*_O_ is the radius of the orbital trajectory. In the tilt mixer, the flow is driven by gravity which yields a lateral acceleration of *g* sin *α*, where *g* represents gravitational acceleration and *α* the tilt angle. To achieve the same lateral acceleration, an orbital shaker must therefore operate at a frequency 
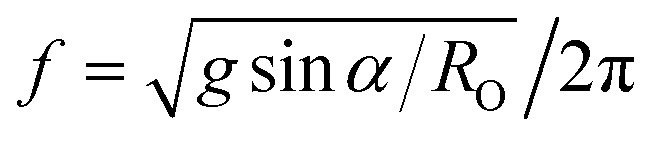
. For an orbital radius of 2.5 cm, this corresponds to a shaker frequency of approximately 1 Hz (or 60 rpm). So if the orbital shaker frequency were doubled to 120 rpm for example, the centripetal acceleration would be quadrupled. In contrast, the lateral acceleration in the tilt mixer remains constant at *g* sin *α* for all mixer speeds. This may help to understand why oscillatory shakers can generate breaking waves at certain speeds, necessitating the identification of optimal operating conditions where no breaking waves are formed, depending on the specific channel dimensions and shaker radius.^10,21^ Furthermore, within the channel the flow and the generated shear stresses using an oscillatory shaker are non-uniform. These can lead to the upregulation of proatherogenic genes in cells at the center of the channel and of more atheroprotective genes in the cells at the channel edge.^22^

In pulsatile-flow culture, the nutating motion of the tilt mixer produces a wave in the well that travels at the same speed as the mixer itself. This direct pulsatile nature of the wave, which consequently generates pulsatile shear stress, closely mimics the physiological pulsatile shear stress experienced in a blood vessel at 60 and 120 beats per minute (corresponding to shaker speeds of 60 and 120 rpm, respectively). This characteristic makes pulsatile-flow culture an appropriate system for studying endothelial cells under conditions that closely resemble physiological states.

In addition to bed shear stresses, the cells in pulsatile-flow culture are also exposed to cyclical stretch and compression parallel to the well plate caused by pulsating hydrostatic pressures in the nutating well. For the given configuration, the peak amplitude of these hydrostatic pressures amounts to approximately 35 Pa. Considering that endothelial cells in blood vessels are exposed to cyclical stretching caused by blood pressures of several thousand Pascal, we believe that the lateral pressures in the nutating mixer have a negligible effect on endothelial cell biology.

Using a laboratory nutating tilt mixer and a standardized composition of cell-culture media, shear-stress calculations (Table 1) at various speeds demonstrate that these stresses can be generated within the physiological range for both venous and arterial endothelial cells. At mixer settings of 60 and 120 rpm (corresponding to peak BSS of 3.5 and 10.5 dyn cm^−2^ at *r* = 12 mm, respectively), we observed that porcine endothelial cells exhibited characteristic responses. Specifically, the cytoskeleton of porcine aortic endothelial cells became polymerized and aligned in the direction of flow under flow conditions, in contrast to static conditions. Furthermore, endothelial cells under flow conditions produced significantly more eNOS compared to those under static conditions, confirming the well-established endothelial response to flow.^23^

The present flow-culture system was designed to offer a low-complexity, highly modular *in vitro* model for applying physiological shear stress to large populations of vascular cells for functional analyses. Instead of relying on traditional pumps and tubing, this system employs a circular inclined plane and gravity to generate recirculating fluid flow. The data presented are based on a nutating laboratory mixer with a measured tilt angle of 6° and variable rotation speeds up to 120 rpm. The platform can accommodate multiple 6-well plates, allowing for experiment scalability. Both the flexibility and the modularity of this design address many of the experimental limitations associated with microchannel models.

Pulsatile-flow culture also facilitates investigations of both mono- and co-cultures of vessel mural cells under physiologically relevant flow conditions and different durations. Additionally, the system's cell capacity enables adequate numbers for downstream analyses, such as immunoblotting, without the need for pooling samples. Integrating a porous, cell-culture-compatible membrane within this system protects flow-sensitive smooth muscle cells from direct exposure to shear stress while still allowing for co-culture scenarios where both cells on opposite sides of the membrane can be studied, and their possible interactions through the membrane can be studied further by sectioning the membrane.

This type of membrane, commonly used in transwell systems under static conditions, allows endothelial cell monocultures to be assessed for cellular barrier integrity using TransEndothelial Electrical Resistance (TEER). The TEER assay is typically employed in studies involving patient-derived endothelial cells, such as those from individuals with neurodegenerative diseases like multiple sclerosis.^24^ In pulsatile-flow culture, the same membrane can also be used in TEER assays with the added benefit of including mural cells, such as smooth muscle cells, alongside endothelial cells. This setup allows both cell types to be exposed to their preferred flow conditions prior to TEER, and provides a more physiologically relevant environment for assessing endothelial function.

Pump-driven flow models typically require larger volumes of flow medium, leading to target molecules in the supernatant being too dilute for direct detection without additional processing steps. In contrast, pulsatile-flow culture uses only 1.5 ml of supernatant per well, increasing the relative concentration of potential target molecules. As demonstrated in Fig. 9, an absorbance assay for the formazan target in the supernatant was successfully performed using the pulsatile-flow culture system.

Additional benefits of the open-channel design of pulsatile-flow culture are the abilities to rapidly add and remove test substances and, due to scalability, to accommodate the testing of multiple substances with a high number of replicates. This makes the system particularly well-suited for drug screening applications.

## Methods

### A. Pulsatile-flow culture

#### a. Apparatus

##### I. Rotational tilt mixer

Experiments were performed using CappRondo 3D mixers with clockwise rotation and a continuous platform tilt angle measured at 6° (AHN Biotechnologie GmbH, Nordhausen, Germany). These mixers were surfaced-cleaned with 70% ethanol prior to being placed in a cell-culture incubator and their continuous operation inside did not disturb any autonomous incubator functions. The standard nibbed-rubber platform liners were replaced with thin silicone sheeting to prevent culture-dish shift during rotation.

##### II. Plates

**Fig. 10:**
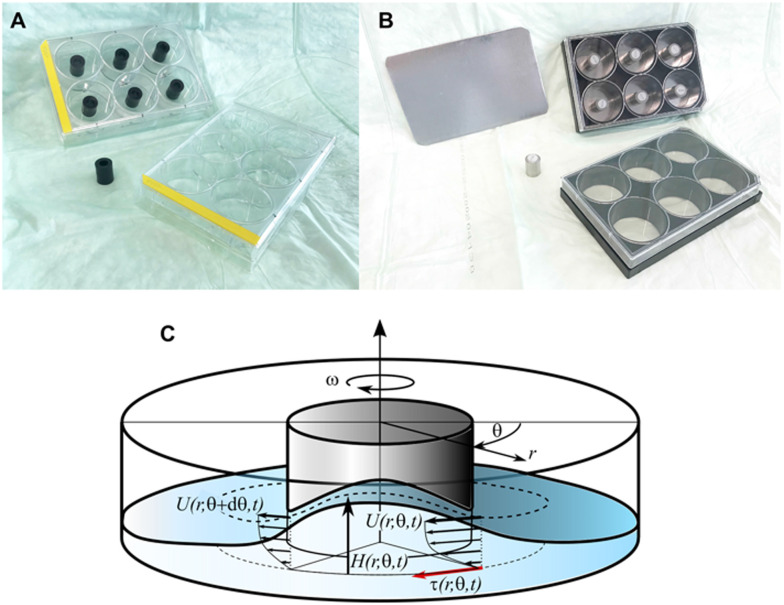
Pulsatile-flow culture apparatus (referred to as the ‘flow channel’ in the text) and its flow characteristics. (A) A plastic cylinder was secured to the center of each well using food-grade epoxy glue. (B) For microscopy, stainless steel cylinders containing a magnet were secured to the inside of each well using a ferrous metal sheet underneath the wells. This setup also allowed a co-culture membranes to be secured to (and easily removed from) the bottom of the well, fixed between the magnetic cylinder and the glass. (C) Schematic of the flow field in the well with coordinates and variables.

Two types of 6-well culture plates were used with the same inside-well dimensions (Fig. 10A and B). The first was a sterile polystyrene plate (Catalog #Z707759, Sigma Aldrich, Switzerland) used for experiments that did not require microscopy. The second type was a sterile plate with glass-bottom wells (Catalog #P06-1.5H-N, Cellvis, Mountain View, CA, USA) appropriate for confocal microscopy.

##### III. Plate centerpieces and configurations

Unidirectional fluid circulation within each well required placement of a hollow centerpiece. The centerpieces were either a stainless steel cylinder (*H* = 15 mm, OD = 12 mm, ID = 6 mm; Wall Stand-off, Tihook, https://Amazon.de) or a polyethylene cylinder of the same dimensions (M6, Enkotrade, https://Amazon.de). For the fixed-center configuration, each cylinder was secured to the center of a well with food-grade adhesive (ET5417, Permabond, Pottstown, PA, USA). The resulting configuration created an open circular channel with an 11.5 mm-wide bed for plating cells. For the removable-center configuration, each cylinder had a neodymium magnet (*H* = 6 mm, OD = 6 mm, The Magnet Shop, Exeter, UK) secured to its center at one end. The stainless steel cylinder had a 10 mm M8 nylon worm screw (Long1688, https://Amazon.de) to secure the magnet, and food-grade epoxy was used for the polyethylene cylinder. This type of magnet/cylinder combination allowed the centerpiece to be held against the glass bottom by attraction to either a non-metal disk (Catalog #GMB297FS, LITKO, Valparaiso, IN, USA) or a thin ferrous metal plate cut to fit the recessed bottoms of the 6-well plates (Fig. 10B). This modular sandwich design also allowed a co-culture barrier membrane (see cell-culture methods below) to be reversibly held in place at the bottom of a well (*i.e.*, between the centerpiece and the glass) for the duration of an experiment. Both magnet-insert types could be reused after cold-clave cleaning (not magnet-damaging autoclaving).

##### IV. Plate preparation for pulsatile-flow culture

Plates were first cold-sterilized using UV light and plasma (CoolClave, Genlantis, San Diego, CA, USA). For endothelial-cell-only cultures, the wells were coated with collagen (Catalog #C4243, Sigma-Aldrich) overnight on a tilt mixer, followed by fibronectin (Catalog #FC010, Sigma-Aldrich) for several hours.

For endothelial cell + smooth muscle cell co-cultures, the wells were not coated. Instead, a circular polyester barrier membrane (12 μm, Catalog #1300017, Sterlitech, Auburn, WA, USA; trimmed to 34 mm OD) was first placed in the bottom of the well and then coated as above with both collagen and fibronectin. This membrane was then used to separate the two cell types (see below) but still allowed for potential cellular communication access through its 0.4 μm pores.

#### b. Measurements of velocity, layer thickness, and shear stress

##### I. Problem definition

To estimate the bed shear stresses (BSS) acting on cells located on the well bed, fluid-layer thicknesses and surface-flow velocities were determined from which BSS could be inferred. To this end, a series of experiments were performed using a customized well filled with 1.5 ml of distilled water at room temperature. This setup had approximately the same fluid density and viscosity as cell-culture medium (*ρ* = 1000 kg m^−3^, *μ* = 0.001 Pa s^−1^). The custom well had the same inner dimensions as a 6-well plate and was fitted with a 12 mm OD centerpiece. It was secured to an LED backlight placed on top of the mixer platform, and a high-speed camera was firmly fixed to the tilting platform to remove any relative motion. The camera images also captured a laser reference marker to synchronize the video frames with respect to the tilt position.

The surface velocity fields of the 1.5 ml fluid were found to have nearly circular streamlines such that the radial components of the velocity vectors could be neglected. The azimuthal velocity components were described by *U*(*r*, *θ*, *t*) as a function of the radial coordinate *r*, the azimuthal angle *θ*, and the time *t* (Fig. 10C). Similarly, fluid-layer thickness was described by the variable *H*(*r*, *θ*, *t*).

##### II. Surface particle image velocimetry

Particle image velocimetry (PIV) was used to quantify the velocity fields on the surface of the fluid moving in the well.^25^ For this, the mixer-induced movements of hollow glass microbeads (Zoic Palaeotech, Sherborne, UK) were recorded at 60 frames per second using a high-speed camera (acA1300-60gm, Basler AG, Ahrensburg, Germany) as they floated on top of the 1.5 ml of distilled water which filled a custom single well (with the same dimensions as channels in a 6-well plate). The original mix of glass microbeads was sifted using a 200 μm cell strainer (Cat #43-50200-03, pluriSelect, Leipzig, Germany) and only the retained beads >200 μm were used. Particle recordings were captured for approximately 10 s yielding 589 image frames for analysis (see Movie S1† for an example).

The recordings of consecutive high-speed images were analyzed using the PIV algorithm implemented in the open-source application PIVlab.^26^ Each consecutive image pair yielded one velocity field. Problems with image quality, uneven particle distribution, and imperfect camera frame rates resulted in outliers that were manually removed from the results. Furthermore, the camera frame rate was insufficient to reliably capture the highest velocities, such that only measurements from image recordings using the 60 rpm mixer speed could be reliably analyzed with PIV. Finally, the velocity fields obtained from independent image pairs were rotated individually (using frame number and angular wave speed) such that the wave form aligned in all velocity fields. These aligned velocity fields were then averaged to obtain an estimate for the surface velocity field.

##### III. Fluid thickness imaging

To determine the fluid layer thickness at different mixer speeds, Evans Blue dye (0.1% w/v, Catalog #E2129, Sigma-Aldrich) was added to the fluid. Under level, static conditions this blue fluid mixture was then added in sequential millimeter-height increments (measured at the channel center) to the custom well described above. At 0 (empty), 1, 2, 3, 4, and 5 mm fluid levels, 10 s recordings (∼589 frames) of LED light transmitted through the fluid were captured, with thin fluid layers resulting in more transmitted light, and less light for thicker fluid layers. The resulting pixel intensities for 0, 1, 2, 3, 4, and 5 mm layer thickness were then used to generate calibration curves for each pixel, which related the individual pixel intensity to the local layer thickness.

These calibration curves were then used to determine the topographies of the pulsatile waveforms of the same fluid (1.5 ml) under dynamic tilt-mixer conditions (60, 80, 100, 110, and 120 rpm). To mitigate measurement noise, results were averaged as outlined above over all frames.

##### IV. Velocity field estimation from fluid layer thickness

As an alternative to the PIV method for measuring surface velocity fields, we developed a method to estimate the surface velocities from the fluid layer thickness. In contrast to PIV, this method was not limited by particle density and frame rate. Therefore, it was used to determine the surface velocities for all configurations. The PIV results obtained for 60 rpm (see above) were used to validate this fluid-layer thickness method.

The presence of a thin fluid layer in the well suggested that shallow-water theory could be applied to study flow in this system. According to this theory, there is a direct relationship between fluid layer thickness (*H*) and the velocity field in the plane of the well based on mass conservation. If a quasi-one-dimensional flow is assumed along circular streamlines with velocity *U* (this observation was supported by the velocity vectors determined with PIV at 60 rpm shown in Fig. 1B), then conservation of mass yields1
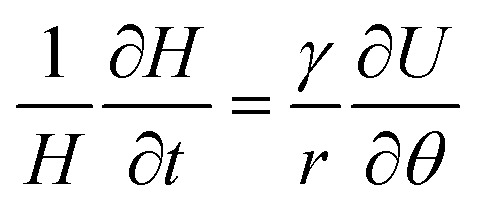
where the parameter *γ* provides a correction for the vertical velocity profile in the fluid layer. For a rectangular velocity profile (as in the classical shallow-water theory) we have *γ* = 1, whereas *γ* = 2/3 for parabolic velocity profiles (for steady viscous flow with a stress-free surface). Because of flow pulsatility, thin Stokes boundary layers were expected in the present experiments, such that we chose *γ* = 1 for a (nearly rectangular velocity profile) in the following calculations (Fig. 1D).

Integration of eqn (1) with respect to azimuthal angle *θ* yields the following expression for the surface velocity *U*2
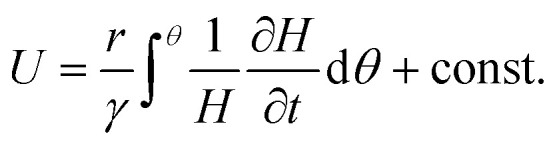
As shown in the results section, fluid flow took the form of a travelling wave that spun around the center of the well at an angular wave speed of *ω*_p_. Therefore, new variables for the material velocity *Û* and fluid layer thickness *Ĥ* of the travelling wave were introduced,

3 *U*(*r*, *θ*, *t*) = *Û*(*r*, *

<svg xmlns="http://www.w3.org/2000/svg" version="1.0" width="11.333333pt" height="16.000000pt" viewBox="0 0 11.333333 16.000000" preserveAspectRatio="xMidYMid meet"><metadata>
Created by potrace 1.16, written by Peter Selinger 2001-2019
</metadata><g transform="translate(1.000000,15.000000) scale(0.011667,-0.011667)" fill="currentColor" stroke="none"><path d="M560 1160 l0 -40 -40 0 -40 0 0 -80 0 -80 40 0 40 0 0 80 0 80 40 0 40 0 0 -80 0 -80 40 0 40 0 0 80 0 80 -40 0 -40 0 0 40 0 40 -40 0 -40 0 0 -40z M320 840 l0 -40 -40 0 -40 0 0 -80 0 -80 -40 0 -40 0 0 -80 0 -80 -40 0 -40 0 0 -200 0 -200 40 0 40 0 0 -40 0 -40 160 0 160 0 0 80 0 80 80 0 80 0 0 120 0 120 40 0 40 0 0 160 0 160 -40 0 -40 0 0 40 0 40 -40 0 -40 0 0 40 0 40 -120 0 -120 0 0 -40z m240 -80 l0 -40 40 0 40 0 0 -80 0 -80 -40 0 -40 0 0 -40 0 -40 -160 0 -160 0 0 80 0 80 40 0 40 0 0 80 0 80 120 0 120 0 0 -40z m0 -440 l0 -80 -40 0 -40 0 0 -40 0 -40 -40 0 -40 0 0 -40 0 -40 -120 0 -120 0 0 160 0 160 200 0 200 0 0 -80z"/></g></svg>

* = *θ* − *ω*_p_*t*)

4 *H*(*r*, *θ*, *t*) = *Ĥ*(*r*, ** = *θ* − *ω*_p_*t*)

where the angle ** moved with the travelling wave. With these definitions, the time derivative of *H* could be rewritten as5
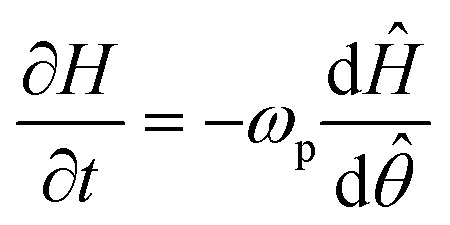
which can be used to solve the integral (2) as6
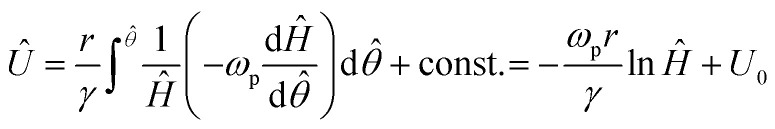
This equation was then used to determine the surface velocities *U* directly from the measured layer height *H* (up to an unknown constant velocity *U*_0_).

##### V. Bed shear stress

To estimate the bed-shear stress *τ* on the bottom of the fluid-filled well, we used the surface velocity *U* obtained from PIV and fluid-layer thickness measurements.

For steady flow (no travelling wave, constant fluid layer thickness), the free-surface velocity *U* drives a parabolic velocity profile *u*(*z*) = *U*(2*zH* − *z*^2^)/*H*^2^ over the fluid layer depth *H* with peak velocity *U* at the stress-free fluid surface, and with zero velocity on the bottom of the well. For this steady configuration, *τ* is given by7
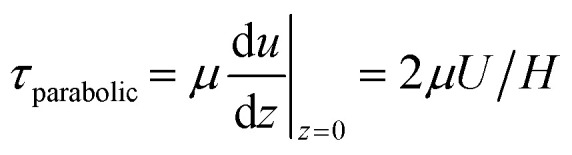
For pulsatile flows, a thin Stokes boundary layer will develop at the bottom of the well, and its thickness can be estimated as8
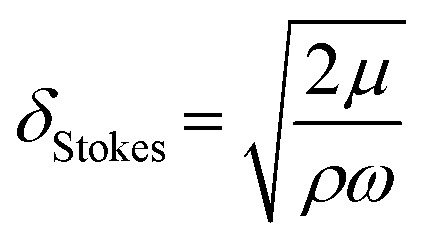
with the characteristic frequency *ω* of the pulsatile flow. For Stokes boundary layers, *τ* is given by9
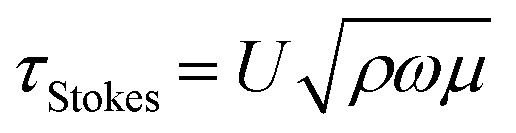
Note that this formula is equivalent to the formula typically used for orbital shakers, 
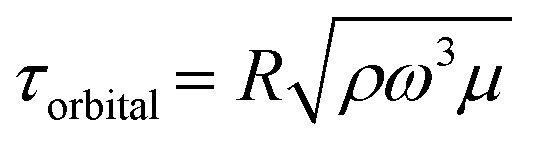
, if we assume that the fluid velocity *U* is equal to the azimuthal velocity of the orbital motion *Rω*, where *R* is the orbital radius and *ω* = 2π*f* is the angular shaker frequency. However, the results indicate that the fluid velocity *U* is different from the azimuthal velocity of the shaker such that the formula for the orbital shaker does not apply in the present model.

### B. Cell culture

#### a. Cells

Cell harvests were conducted in accordance with University of Bern policies for *ex vivo* tissue use. Endothelial cells were harvested under aseptic conditions from fresh *ex vivo* specimens of Landrace pig aorta, vena cava, and coronary arteries. Mural muscle cells were also harvested from the same coronary arteries and thoracic vena cava.

##### I. Endothelial cells

For endothelial cells, harvested cells were swabbed from vessel interiors and then introduced into precoated (fibronectin) 6-well plates with M199 medium supplemented with 10% FBS, 1% penicillin/streptomycin (Catalog #15140122, Thermo Fisher Scientific), 1% l-glutamine (Catalog #25030081, Thermo Fisher Scientific), and 1% endothelial-cell growth factor (Catalog #E9640, Sigma-Aldrich). Cells were allowed to settle and proliferate until confluent in a 5% CO_2_ atmosphere incubator at 37 °C. This was accompanied by daily assessment/removal of fibroblast contamination and change of media. At confluency, these cells were enzymatically released, transferred to larger flasks, and returned to the incubator with the media changed every two days until confluency. These passage 2 (P2) cells were then counted and stored at −150 °C as aliquots containing one million cells.

##### II. Smooth muscle cells

For smooth muscle cells, small sheets of the media layer were stripped from the luminal surface of coronary vessels after endothelial cells were removed, cut into smaller segments (∼1 mm) and collected in M199 media supplemented with 20% FBS and 1% penicillin/streptomycin. These pieces were then spaced evenly onto dry, sterile, uncoated culture plates and allowed to attach by dry adhesion for 10 minutes before being covered by supplemented M199 media and placed in an incubator. Media changes every 2 days resulted in the eventual outflow of migratory smooth muscle cells and progenitors by ∼10 days. The original muscle strips were then removed, and the remaining migratory cells were allowed to become confluent in the incubator. At confluency, these cells were transferred to larger flasks and returned to the incubator with the media changed every two days until confluency. These P2 cells were then counted, characterized and stored at −150 °C as aliquots containing 500 000 cells.

#### b. Culture conditions

##### I. Monoculture

For flow-culture experiments involving only endothelial cells, P2 cells were thawed, expanded in complete M199 media supplemented with growth factor (Catalog #E9640, Sigma-Aldrich) until 90% confluency and then were plated into collagen/fibronectin-coated wells at 40 000 cells per cm^2^. After 24 hours of incubation under static conditions (37 °C, 5% CO_2_), cells were incubated for 48 hours under static or flow conditions (60 rpm or 120 rpm). For evaluation of heparan sulfate expression, after 48 hours of respective flow conditions, endothelial cells were treated with 2 U ml^−1^ heparinase I + III for 2 hours (Sigma-Aldrich, Catalog #H3917-100UN).

##### II. Co-culture

For co-culture experiments on porous membranes, P2 smooth muscle cells were thawed, expanded in complete M199 media until 90% confluency and then 20 000 cells per cm^2^ were applied through a 100 μm strainer (pluriSelect, Catalog #435010003) to a pre-coated barrier membrane (Sterlitech, Catalog #1300017) and covered with the same media. After a static period of 24 hours at 37 °C and 5% CO_2_, the combination of membrane + muscle cells was inverted (cells facing down), and covered in the same media. In the next step, endothelial cells (P3) were seeded on the same membrane at 40 000 cells per cm^2^ facing up. After 24 hours of static conditions at 37 °C and 5% CO_2_, the membrane was fixed in a glass-bottom well plate using a magnetic centerpiece and a ferrous disk. Endothelial cells were subjected to either flow or static conditions for an additional 48 hours.

For both co-culture and monoculture experiments under flow, the mixer speed started at 60 rpm and then was gradually increased in increments of 20 rpm per hour until the final desired speed was reached.

### C. Cellular function analyses

#### a. Immunofluorescent staining

Cells were fixed inside the wells with 4% paraformaldehyde in PBS, washed with PBS, and then permeabilized with PBS + 0.1% Triton X-100. After blockade of non-specific binding using 3% BSA in PBS, the cells were incubated (overnight, 4 °C) with primary antibodies against:

• CD31 (1 : 200, Catalog #MAB33871, R&D System).

• eNOS (1 : 100, Catalog #Bs-0163R, Bioss).

• Alpha smooth muscle actin (α-SMA) (1 : 300, Catalog #Ab7817, Abcam).

• Heparan sulfate (1 : 300, Catalog #370255, Amsbio).

After washing the cells with PBS, they were incubated for one hour at room temperature with appropriate secondary antibodies labeled with different colored fluorophores. After additional PBS washes, the cells were imaged using an inverted Zeiss LSM980 confocal microscope.

#### b. Immunoblot analysis of eNOS expression

P2 aortic endothelial cells were thawed, expanded in supplemented M199 media until confluency, and then plated into collagen/fibronectin coated wells at 40 000 cells per cm^2^. For 48 hours the cells were then cultured under static or flow (120 rpm) conditions in a 5% CO_2_, 37 °C incubator. The cells were then washed briefly with PBS after media removal and then lysed for 30 minutes on ice using RIPA buffer (Catalog #89901, Thermo Fisher Scientific, Zurich, Switzerland) containing protease inhibitors (Halt Inhibitor Cocktail, Catalog #78444, Thermo Fisher Scientific) and the soluble proteins collected after centrifugation to remove cellular debris. Aliquots of stock solutions were frozen at −20 °C to limit freeze–thaw degradation. After protein quantitation (Pierce BCA kit, Catalog #23227, Thermo Fisher Scientific) using bovine serum albumin as a standard, proteins were separated using 4–12% Bis-Tris gels (BOLT, Catalog #NW04120BOX, Thermo Fisher Scientific) and transferred to PVDF membranes (iBlot2 Transfer Stack, Catalog #IB24001 and Catalog #IB23001, Thermo Fisher Scientific). For protein-level normalization, all membrane proteins were stained using TotalStain Q (Catalog #AC2225, Azure Biosystems, Dublin, CA) and UV-imaged using a Quantum CX5 imager (Vilber Lourmat, Eberhardzell, Germany). The membranes were blocked for non-specific staining using 1% bovine serum albumin for 30 minutes and then incubated for 30 minutes with a primary antibody against eNOS (1 : 5000, Catalog #610297, BD Biosciences, Allschwil, Switzerland). After washing with tris-buffered saline + 0.05% Tween, the blots were incubated for 30 minutes with an appropriate secondary antibody (1 : 20 000, Catalog #115-035-003, Jackson ImmunoResearch, Cambridgeshire, UK). After additional washing, the blots were developed using chemiluminescence (WesternBright ECL, Catalog #K-12045, Advansta, San Jose, CA) and imaged using a Fusion FX7 imager (Vilber Lourmat).

#### c. Cell viability absorbance assay

Monocultures of endothelial cells at an initial density of 20 000 cells per cm^2^, as described in section C, were prepared. After 48 hours of either flow (60 rpm) or static conditions, a 1% solution of tetrazolium salt (Sigma-Aldrich, Catalog #05015944001) in complete M199 media was added to each well. The channels were then maintained under their respective conditions for an additional 4 hours. Following this incubation period, the cell supernatants were collected, and 450 nm absorbance measurements were made using a microplate reader (ThermoFisher, Varioskan LUX Multimode Microplate Reader). Measurements were adjusted by subtracting background absorbance values and then normalized as a viability index with static-condition values set to 1 for statistical comparison.

### D. Statistical analysis

Unless otherwise noted, all data are represented by mean ± SD. Prism software (v. 10.2.3, GraphPad Software, Boston, MA, USA) was used to perform *t*-tests, and a one-way ANOVA for comparing the three flow conditions. A *p* < 0.05 level was considered significant.

## Conclusion

This novel, robust, and economical system presents solutions for many of the difficulties encountered by users of microfluidic channels to study structural cells of blood vessels under flow conditions. It also offers the possibility of extending experimental observations from mono- to co-cultures. Future applications of this system under current development include assays where blood components are added to such cultures to assess interactions under flow conditions (*e.g.*, clotting, neutrophil rolling, migration of peripheral blood mononuclear cells and muscle-cell migration).

## Data availability

The data that support the findings of this study are available from the corresponding author upon reasonable request.

## Author contributions

NS-A: designed and performed experiments, analyzed data, prepared manuscript. RR: supervised project, edited manuscript. DO: analyzed data, edited manuscript.

## Conflicts of interest

There are no conflicts to declare.

## Supplementary Material

LC-025-D4LC00949E-s001

LC-025-D4LC00949E-s002

LC-025-D4LC00949E-s003

LC-025-D4LC00949E-s004

LC-025-D4LC00949E-s005

## References

[cit1] Galley H. F., Webster N. R. (2004). Br. J. Anaesth..

[cit2] Mylvaganam S., Plumb J., Yusuf B., Li R., Lu C.-Y., Robinson L. A., Freeman S. A., Grinstein S. (2022). Nat. Cell Biol..

[cit3] Davies P. F. (1995). Physiol. Rev..

[cit4] Sfriso R., Zhang S., Bichsel C. A., Steck O., Despont A., Guenat O. T., Rieben R. (2018). Sci. Rep..

[cit5] Roknujjaman Md., Kyotoh H., Yohei A., Yasuhisa A. (2023). Phys. Fluids.

[cit6] Yang Y., Fathi P., Holland G., Pan D., Wang N. S., Esch M. B. (2019). Lab Chip.

[cit7] Wong K. H. K., Chan J. M., Kamm R. D., Tien J. (2012). Annu. Rev. Biomed. Eng..

[cit8] Busek M., Aizenshtadt A., Koch T., Frank A., Delon L., Martinez M. A., Golovin A., Dumas C., Stokowiec J., Gruenzner S., Melum E., Krauss S. (2023). Lab Chip.

[cit9] Fernandes A., Hosseini V., Vogel V., Lovchik R. D. (2022). PLoS One.

[cit10] Warboys C. M., Ghim M., Weinberg P. D. (2019). Atherosclerosis.

[cit11] Alpresa P., Sherwin S., Weinberg P., van Reeuwijk M. (2018). Phys. Fluids.

[cit12] Wang C., Baker B. M., Chen C. S., Schwartz M. A. (2013). Arterioscler., Thromb., Vasc. Biol..

[cit13] Leo F., Suvorava T., Heuser S. K., Li J., LoBue A., Barbarino F., Piragine E., Schneckmann R., Hutzler B., Good M. E., Fernandez B. O., Vornholz L., Rogers S., Doctor A., Grandoch M., Stegbauer J., Weitzberg E., Feelisch M., Lundberg J. O., Isakson B. E., Kelm M., Cortese-Krott M. M. (2021). Circulation.

[cit14] Yu J., Zhang Y., Zhang X., Rudic R. D., Bauer P. M., Altieri D. C., Sessa W. C. (2012). PLoS One.

[cit15] Milusev A., Despont A., Shaw J., Rieben R., Sorvillo N. (2023). Sci. Rep..

[cit16] Alkazemi H., Mitchell G. M., Lokmic-Tomkins Z., Heath D. E., O'Connor A. J. (2023). ACS Appl. Mater. Interfaces.

[cit17] Alkazemi H., Huang T., Mail M., Lokmic-Tomkins Z., Heath D. E., O'Connor A. J. (2023). ACS Appl. Mater. Interfaces.

[cit18] Rich P. R., Mischis L. A., Purton S., Wiskich J. T. (2001). FEMS Microbiol. Lett..

[cit19] Frydrychowicz A., Stalder A. F., Russe M. F., Bock J., Bauer S., Harloff A., Berger A., Langer M., Hennig J., Markl M. (2009). J. Magn. Reson. Imaging.

[cit20] Uzarski J. S., Scott E. W., McFetridge P. S. (2013). PLoS One.

[cit21] Thomas J. M. D., Chakraborty A., Berson R. E., Shakeri M., Sharp M. K. (2017). AIChE J..

[cit22] Dardik A., Chen L., Frattini J., Asada H., Aziz F., Kudo F. A., Sumpio B. E. (2005). J. Vasc. Surg..

[cit23] Basehore S. E., Bohlman S., Weber C., Swaminathan S., Zhang Y., Jang C., Arany Z., Clyne A. M. (2021). Circ. Res..

[cit24] Nishihara H., Perriot S., Gastfriend B. D., Steinfort M., Cibien C., Soldati S., Matsuo K., Guimbal S., Mathias A., Palecek S. P., Shusta E. V., Pasquier R. D., Engelhardt B. (2022). Brain.

[cit25] Raffel M. , Willert C. E., Scarano F., Kähler C. J., Wereley S. T. and Kompenhans J., Particle Image Velocimetry: A Practical Guide, Springer International Publishing, Cham, 2018

[cit26] Thielicke W., Stamhuis E. J. (2014). J. Open Res. Softw..

