# Supplementary material for: Pulsatile-flow culture: a novel system for assessing vascular-cell dynamics
Source: Lab Chip. 2025 Feb 24;25(7):1755–66. doi: 10.1039/d4lc00949e (PMC11869938; doi:10.1039/d4lc00949e)
Supplement: LC-025-D4LC00949E-s001 [file LC-025-D4LC00949E-s001.pdf]

Supplementary Information (SI) for Lab on a Chip.
This journal is © The Royal Society of Chemistry 2025

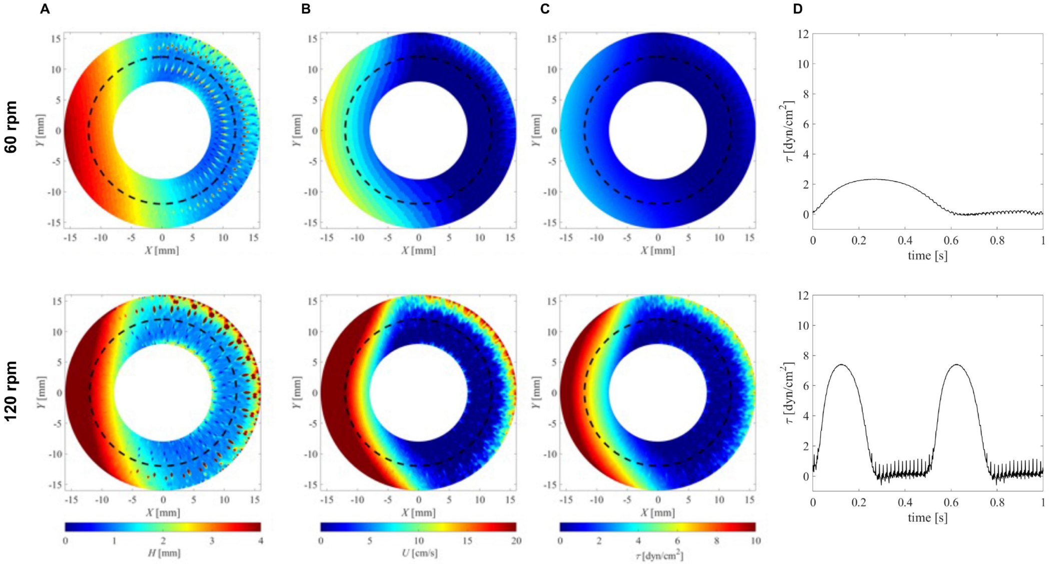

Fig. S1 Examples of flow-culture dynamics at two different rotational speeds for the low viscosity medium ($\mu = 1\ mPa * s$). (A) Heat maps of fluid layer thickness H. (B) Heat maps of surface velocity U. (C) Heat maps of bed shear stress fields τ. (D) Waveform of bed shear stress at a radius of 12 mm (corresponding to dashed circular line in A, B, C).
