# Supplementary material for: Pulsatile-flow culture: a novel system for assessing vascular-cell dynamics
Source: Lab Chip. 2025 Feb 24;25(7):1755–66. doi: 10.1039/d4lc00949e (PMC11869938; doi:10.1039/d4lc00949e)
Supplement: LC-025-D4LC00949E-s002 [file LC-025-D4LC00949E-s002.pdf]

Supplementary Information (SI) for Lab on a Chip.
This journal is © The Royal Society of Chemistry 2025

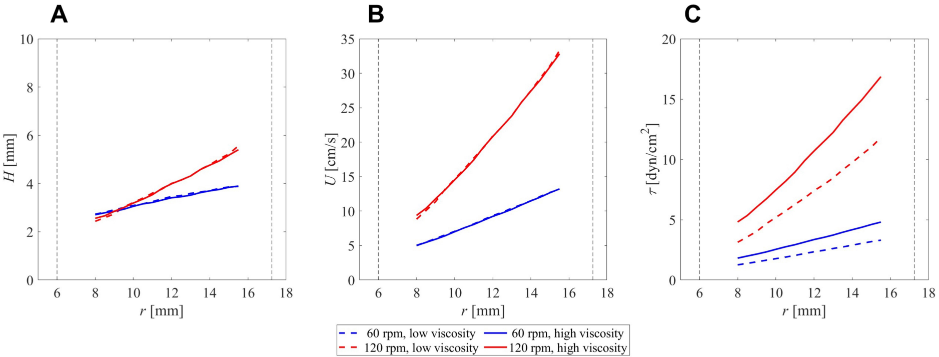

Fig. S2 Media-wave profiles across cross-sections of pulsatile-flow culture channels using different mixer speeds and viscosities. (A) Media heights across the channel, showing minimal variability between high-viscosity and low-viscosity media. (B) Media-surface speeds were observed to be nearly double at each point for high-viscosity media compared to low-viscosity media. (C) Translated bed shear stresses derived from surface speeds from channel cross-sections. Shear stresses exhibited the greatest variations at higher speeds, particularly in the high-viscosity media.
