# Supplementary material for: Pulsatile-flow culture: a novel system for assessing vascular-cell dynamics
Source: Lab Chip. 2025 Feb 24;25(7):1755–66. doi: 10.1039/d4lc00949e (PMC11869938; doi:10.1039/d4lc00949e)
Supplement: LC-025-D4LC00949E-s003 [file LC-025-D4LC00949E-s003.pdf]

Supplementary Information (SI) for Lab on a Chip.
This journal is © The Royal Society of Chemistry 2025

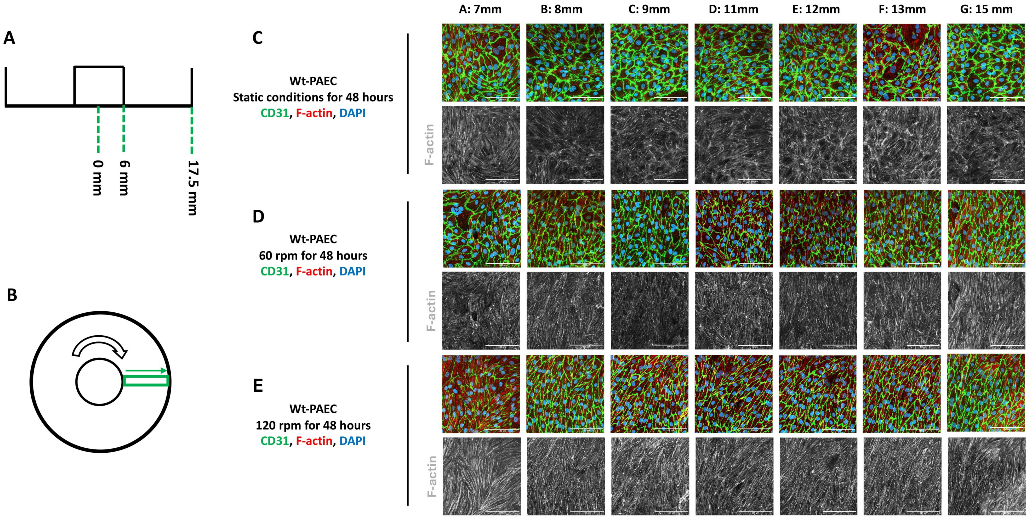

Fig. S3 Images from different cross-section locations of a pulsatile-flow culture channel under various flow conditions, and assessment of F-actin directionality. (A) Pulsatile-flow culture channel cross-section with its dimension. (B) Flow direction in the representative images under flow conditions and the location of images. (C) Representative images (n = 7) of endothelial cells within a channel cross-section under static conditions. Cells exhibited random alignment with no discernible pattern. (D) Endothelial cells under 60 rpm flow conditions experienced low shear stresses, resulting in reduced variability in their alignment and directionality. (E) Endothelial cells under 120 rpm flow conditions experienced higher shear stresses across the channel, leading to slightly more diverse cytoskeletal (F-actin) orientations.
