# Supplementary material for: Pulsatile-flow culture: a novel system for assessing vascular-cell dynamics
Source: Lab Chip. 2025 Feb 24;25(7):1755–66. doi: 10.1039/d4lc00949e (PMC11869938; doi:10.1039/d4lc00949e)
Supplement: LC-025-D4LC00949E-s004 [file LC-025-D4LC00949E-s004.pdf]

Supplementary Information (SI) for Lab on a Chip.
This journal is © The Royal Society of Chemistry 2025

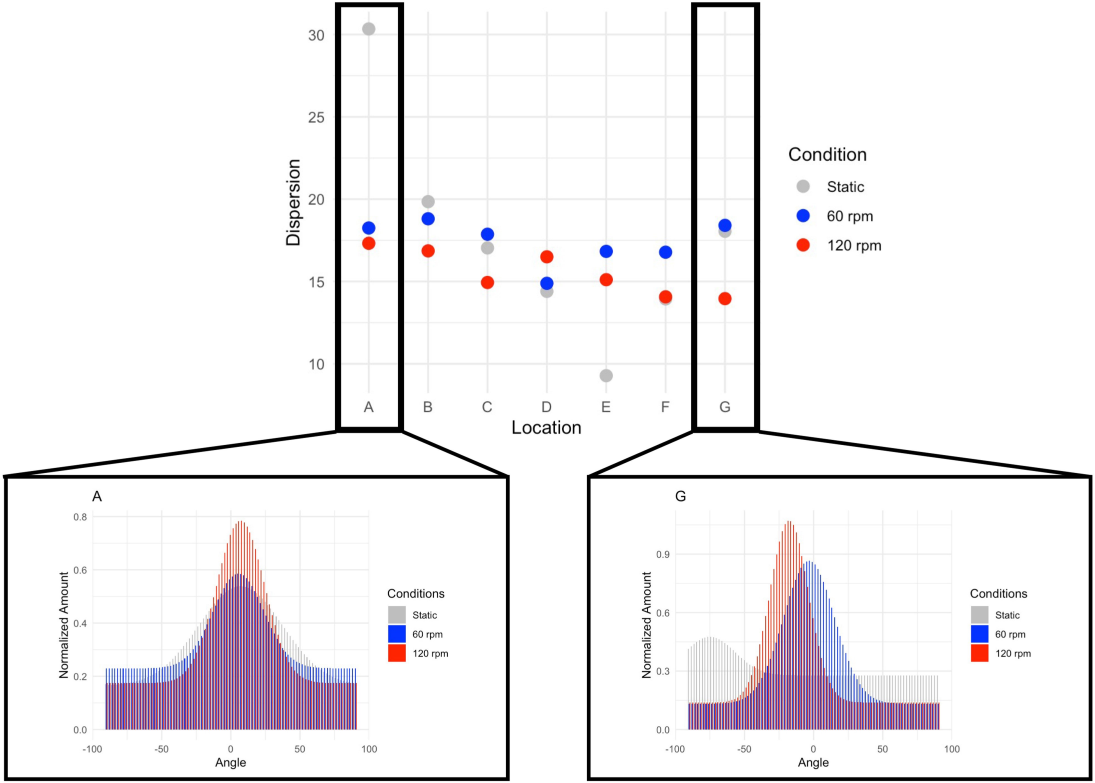

Fig. S4 Alignment dispersion of cytoskeletal molecule F-actin at each radial location across the channel cross-section. Endothelial cells under 120 rpm flow conditions exhibited lower dispersion of F-actin alignment, indicating a stronger alignment along a dominant angle. Cells under 60 rpm flow conditions displayed higher dispersion, reflecting greater variability in alignment. Under static conditions, F-actin directionality was nonspecific, with no dominant alignment observed.
